# Supplementary material for: Confinement of excited states in two-dimensional, in-plane, quantum heterostructures
Source: Nat Commun. 2024 Jul 28;15:6361. doi: 10.1038/s41467-024-50653-x (PMC11284221; doi:10.1038/s41467-024-50653-x)
Supplement: Supplementary file 1 — Supplementary Information [file 41467_2024_50653_MOESM1_ESM.pdf]

Supplementary Information

Confinement of Excited States in Two-Dimensional, In-Plane, Quantum Heterostructures

Gwangwoo Kim[1,2], Benjamin Huet[3], Christopher E. Stevens[4,5], Kiyoung Jo[1], Jeng-Yuan Tsai[6], Saiphaneendra Bachu[7], Meghan Leger[7], Seunguk Song[1], Mahfujur Rahanman[1], Kyung Yeol Ma[8], Nicholas R, Glavin[9], Hyeon Suk Shin[10,11], Nasim Alem[3,7], Qimin Yan[6], Joshua R. Hendrickson[4], Joan M. Redwing[3,7], Deep Jariwala[1,]*

[1]Department of Electrical and Systems Engineering, University of Pennsylvania, Philadelphia, PA 19104, USA.

[2]Department of Engineering Chemistry, Chungbuk National University, Chungbuk, 28644, Republic of Korea.

[3]2D Crystal Consortium-Materials Innovation Platform, Materials Research Institute, The Pennsylvania State University, University Park, PA 16802, USA.

[4]Air Force Research Laboratory, Sensors Directorate, Wright-Patterson Air Force Base, OH 45433, USA.

[5]KBR Inc., Beavercreek, OH 45431, USA.

[6]Department of Physics, Northeastern University, Boston, Massachusetts 02115, USA.

[7]Department of Materials Science and Engineering, The Pennsylvania State University, University Park, PA 16802 USA

[8]*Department of Chemistry, Ulsan National Institute of Science and Technology (UNIST), UNIST-gil 50, Ulsan 44919, Republic of Korea.*

[9]*Air Force Research Laboratory, Materials and Manufacturing Directorate, Wright-Patterson Air Force Base, OH 45433, USA.*

[10]*Department of Energy Science and Department of Chemistry, Sungkyunkwan University (SKKU), Suwon 16419, Republic of Korea.*

[11]*Center for 2D Quantum Heterostructures, Institute of Basic Science (IBS), Sungkyunkwan University (SKKU), Suwon 16419, Republic of Korea*

***Corresponding Authors**

E-mail Addresses: dmj@seas.upenn.edu

Supplementary Figure S1

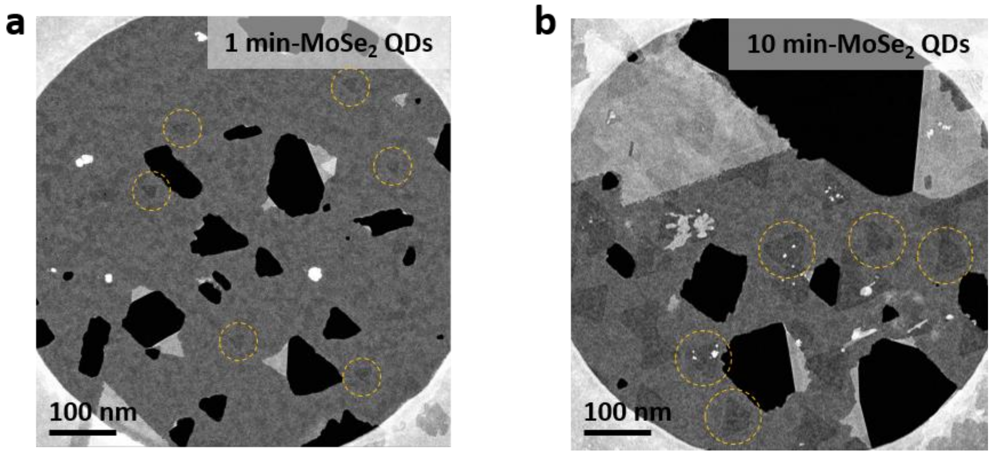

Figure S1. Comparison of the size of the $MoSe_2$ quantum dots (QDs) in the heterostructures with different growth times of $MoSe_2$ QDs. (**a**, **b**) Low magnification annual dark-field scanning TEM (ADF-STEM) images of the heterostructures with the $MoSe_2$ QDs (**a**: 1 min, **b**: 10 mins).

Supplementary Figure S2

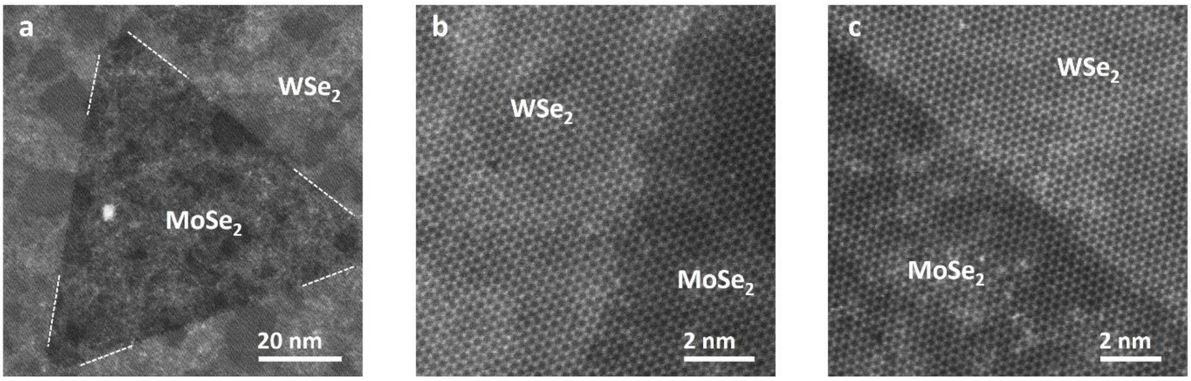

Figure S2. (**a**) ADF-STEM image of the heterostructures with the 10 min-$MoSe_2$ QDs. (**b**, **c**) Atomic-resolution ADF-STEM image showing an interface between the $MoSe_2$ QDs and the WSe_2 matrix.

Supplementary Figure S3

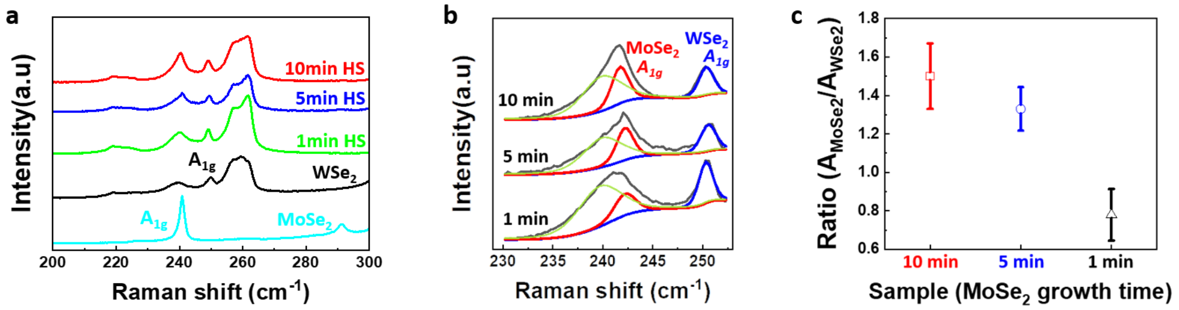

Figure S3. Raman comparison on the heterostructures with different growth times (1, 5, 10 min) of $MoSe_2$ QDs. (**a**, **b**) Raman spectra of the heterostructures (10 min: red, 5 min: blue, 1 min: green), WSe_2 (black) and $MoSe_2$ monolayers (sky blue). Figure **b** is magnified in the range of 230-255 cm^{-1} in Figure **a**, and each peak is deconvoluted. (**c**) Comparison of Raman area ratio of $MoSe_2$ A1g mode to WSe_2 A1g mode on the heterostructures.

Supplementary Figure S4

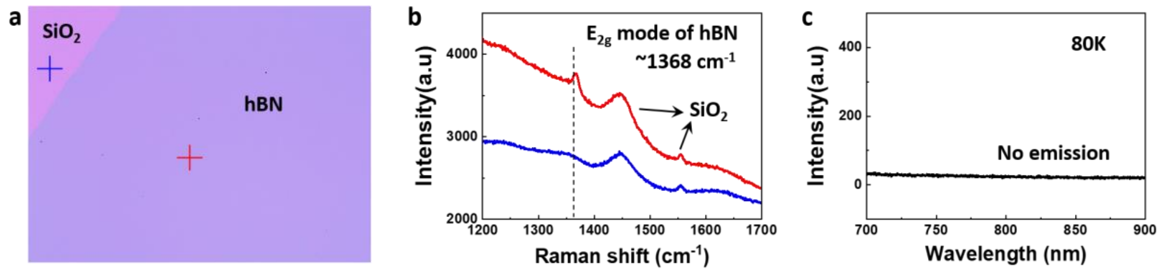

Figure S4. Characterization of h-BN tri-layers used for encapsulation. (**a**) Optical microscopic image of the h-BN tri-layers transferred on a SiO_2 substrate. (**b**) Raman spectra of the h-BN film (red) and the bare SiO_2 substrate (blue) using a 633 nm-laser. The measured points are marked with red and blue crosses in Figure **a**. (**c**) Photoluminescence (PL) spectrum of the h-BN film on SiO_2 substrate, which was measured at 80 K.

Supplementary Figure S5

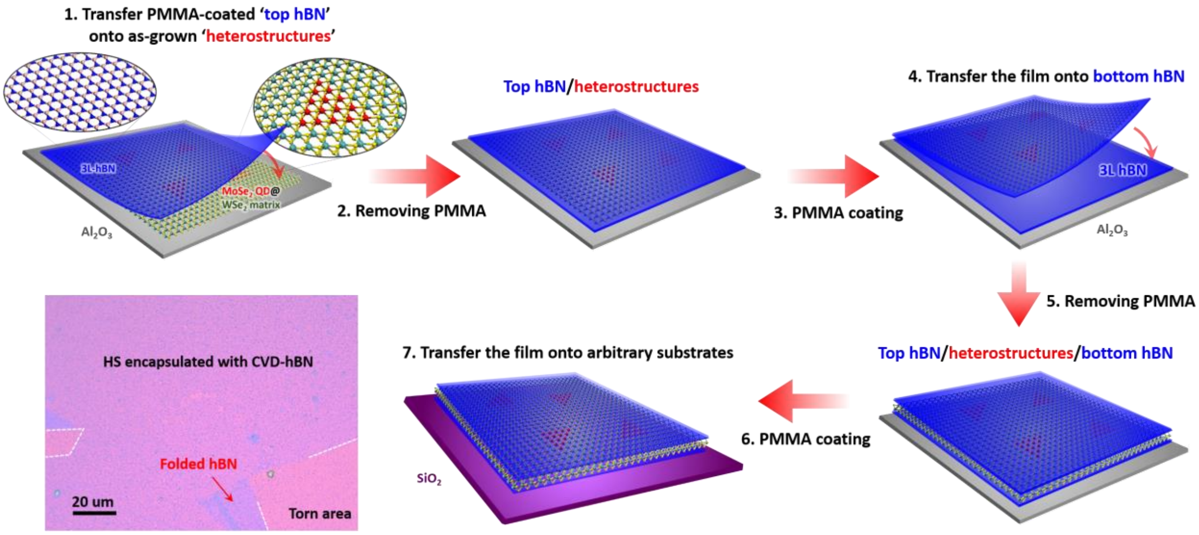

Figure S5. Fabrication process of the $MoSe_2$ QDs@WSe_2 heterostructures encapsulated in top and bottom h-BN layers. (**Bottom left**) An optical microscopic image of the quantum heterostructures encapsulated with h-BN tri-layers, transferred on the SiO_2 substrate.

Supplementary Figure S6

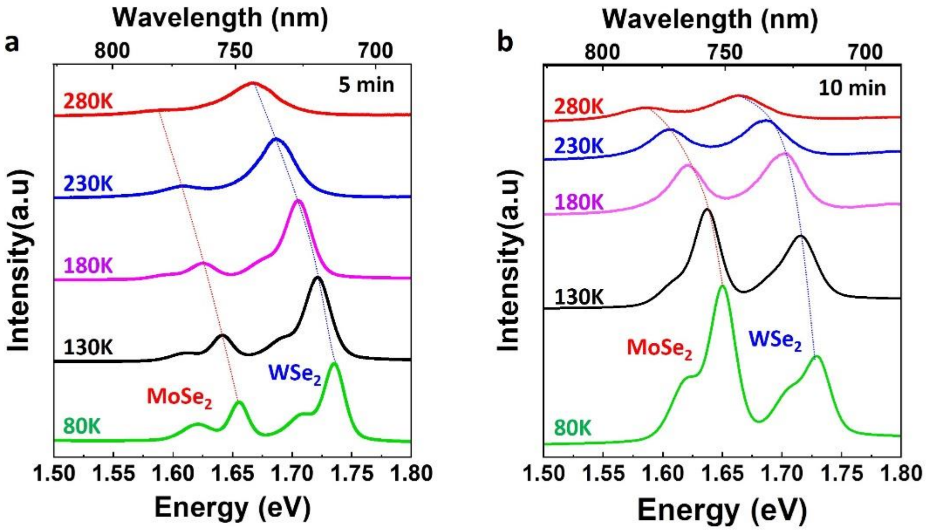

Figure S6. Temperature-dependent PL spectra (280 K to 80 K) of the heterostructures with different growth times of the $MoSe_2$ QDs (**a**: 5 min, **b**: 10 min). These spectra were obtained by a 633 nm CW laser with an excitation power of 20 μW and a 50x lens with 0.35 NA.

Supplementary Figure S7

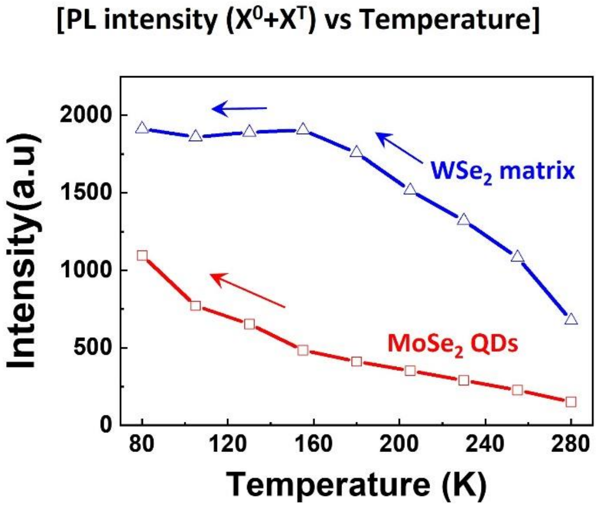

Figure S7. PL intensity change of $MoSe_2$ QDs (red) and WSe_2 (blue) excitons on a 5 min-$MoSe_2$ QDs heterostructures as a function of temperature. The points were plotted from the spectra in **Figure 3b** and **3c**.

Supplementary Figure S8

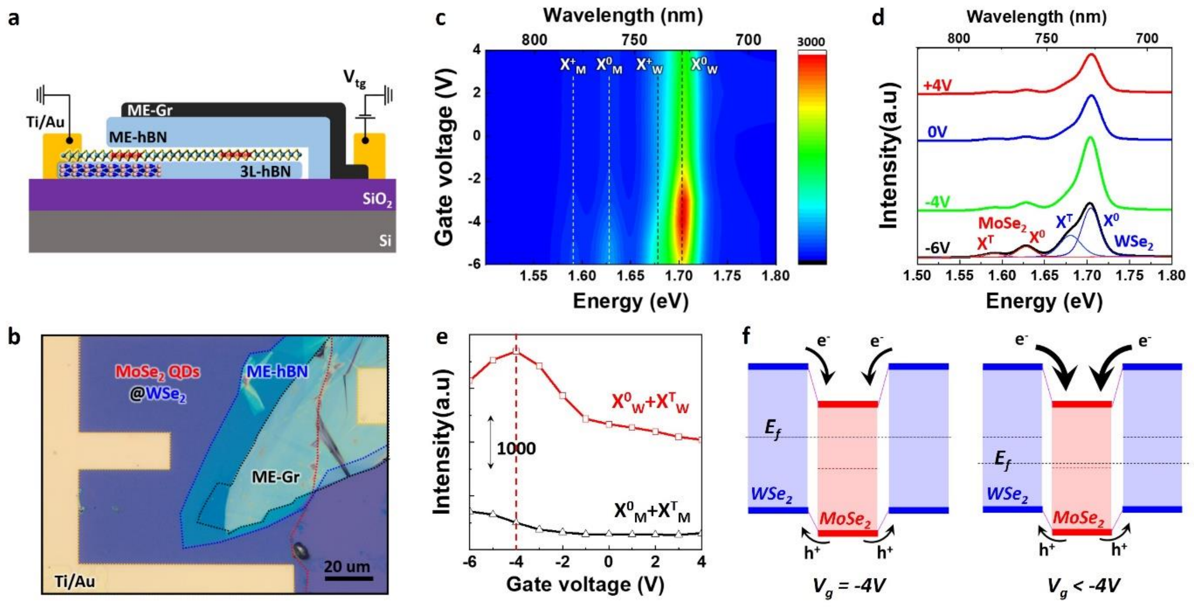

Figure S8. Gate-dependent PL spectroscopy of the quantum heterostructures. (**a**, **b**) Schematic (**a**) and optical microscopic image (**b**) of the h-BN-encapsulated quantum heterostructures device with h-BN dielectric layers and graphene top gate electrode. (**c**) PL spectra of the heterostructures at 80 K as a function of the top gate voltage. The color represents the PL intensity. (**d**) PL spectra at the top gate voltage of +4, 0, -4 and -6 V. As the gated voltage sweep decreased from +4V to -6V, there was a corresponding increase in the intensity of $MoSe_2$ and WSe_2 trion observed on the heterostructure, indicating the presence of positively charged trion (X^+). (**e**) PL intensity change of WSe_2 (red) and $MoSe_2$ (blue) PL peaks as a function of gate voltage. Remarkably, the WSe_2 signal decreased below -4V gate voltage, indicating that the Fermi level was in the middle of the WSe_2 bandgap at a bias of -4 V. However, if a lower bias than this was applied, the signal became smaller due to Pauli blocking as it approached the valence band. (**f**) Schematic band diagram of the quantum heterostructures with the Fermi level modulated by electrostatic gate voltage (V_g). The difference in the conduction band edge between the two materials was much larger than that of the valence band. With a decrease in the Fermi level, more electron transfer was induced, leading to further changes in the PL spectra.

Supplementary Figure S9

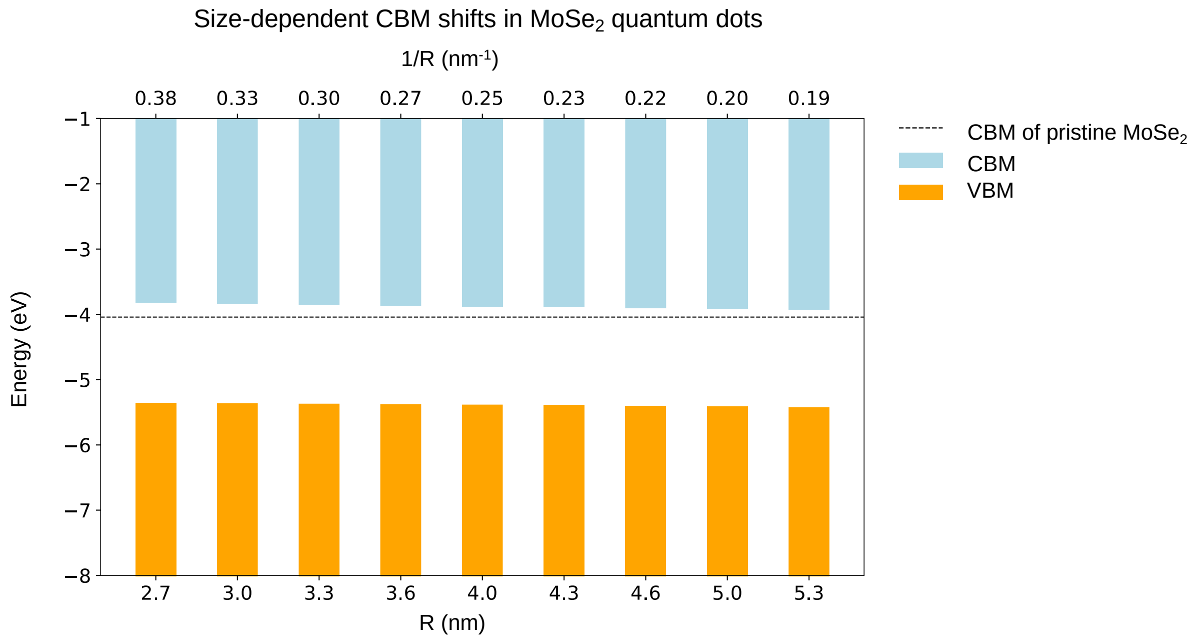

Figure S9. Size-dependent shift of the conduction band minima (CBM) of $MoSe_2$ QDs embedded in WSe_2. The cyan and orange bars represent the CBM and the valence band maxima (VBM), respectively. The edge lengths of $MoSe_2$ QDs range from 2.7 to 5.3 nm. The dashed line represents the CBM of pristine $MoSe_2$, which is located at -4.04 eV below the vacuum energy.

Supplementary Figure S10

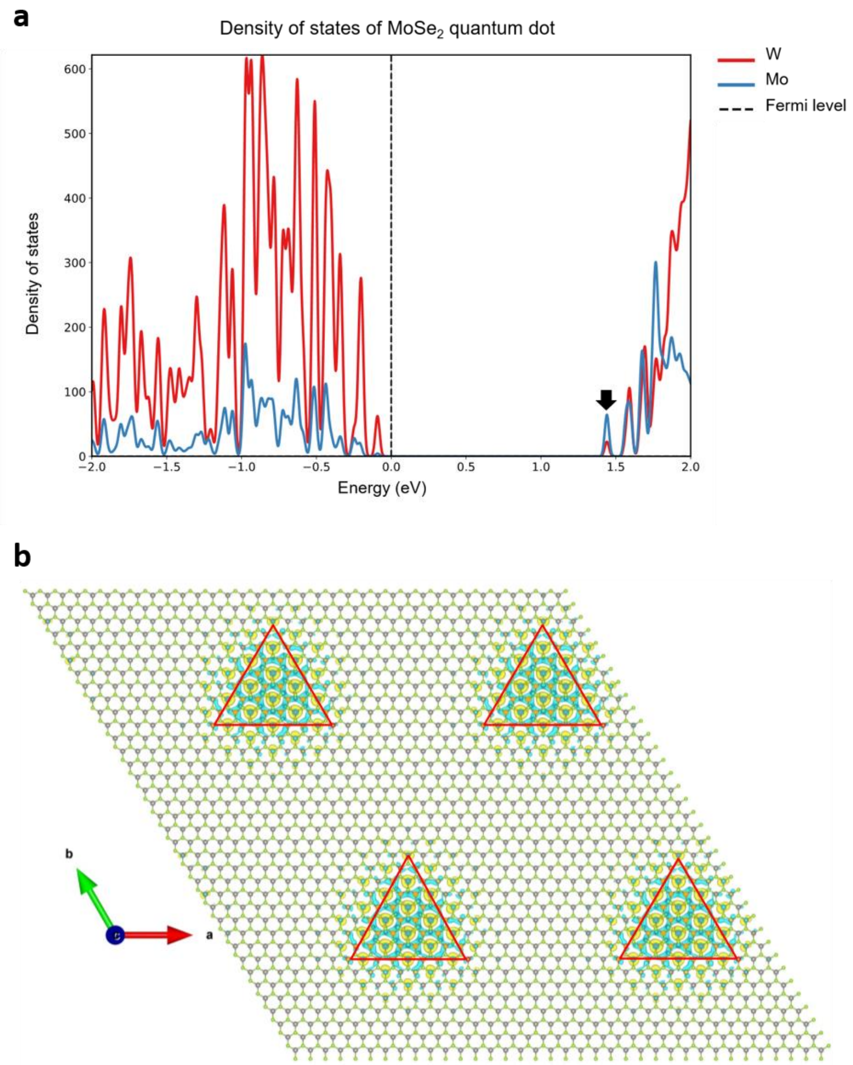

Figure S10. Projected density of states and the wave function of electronic state at the CBM in a 2.7-nm $MoSe_2$ QDs embedded in an 18x18 supercell of WSe_2. (**a**) The projected density of states showing the contributions of Mo and W (represented by blue and red lines, respectively). The black arrow indicates the CBM. (**b**) The wave function of the electronic state at the CBM exhibits a strong localization within the $MoSe_2$ QDs denoted by the red triangle.

Supplementary Figure S11

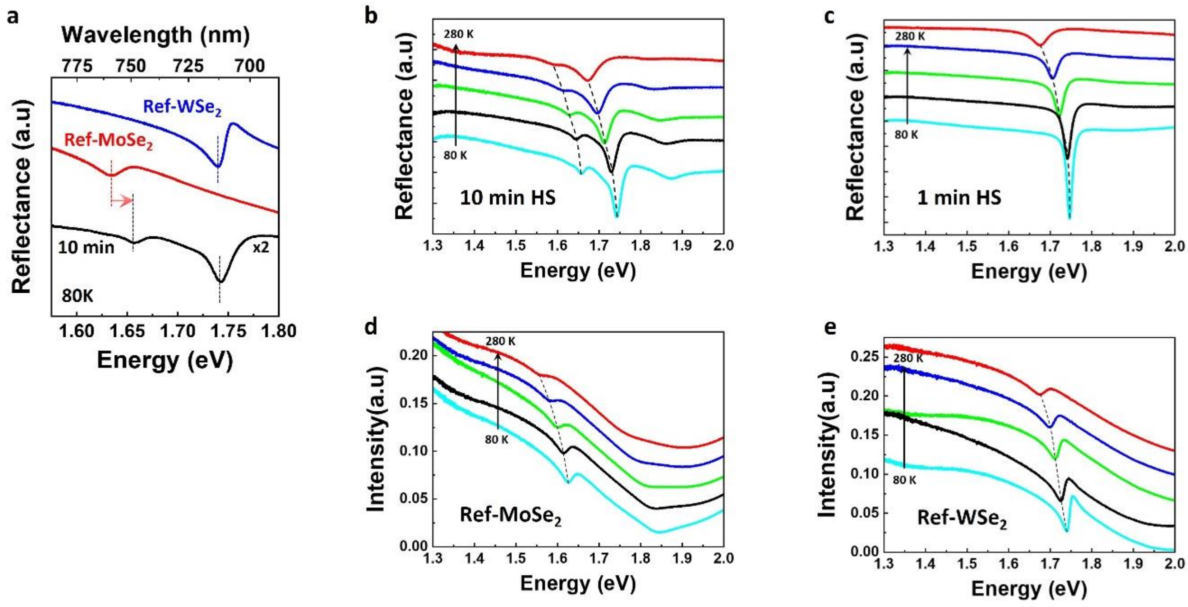

Figure S11. Temperature-dependent reflectance spectra (280 K to 80 K) of the heterostructures (b: 10 min-$MoSe_2$ QDs, c: 1 min-$MoSe_2$ QDs) and reference monolayers (d: $MoSe_2$, e: WSe_2 monolayers). (**a**) Reflectance spectra of the heterostructures (10 min-$MoSe_2$ QD: black) and reference monolayers ($MoSe_2$: red, WSe_2: blue) measured at 80 K. The references $MoSe_2$ and WSe_2 monolayers are prepared in the same metal organic chemical vapor deposition (MOCVD) chamber. For the 1 min-heterostructure (**c**), the absorption peak of $MoSe_2$ QDs could not be obtained due to the low detection level, similar to PL analysis (**Supplementary Figure S10**).

Supplementary Figure S12

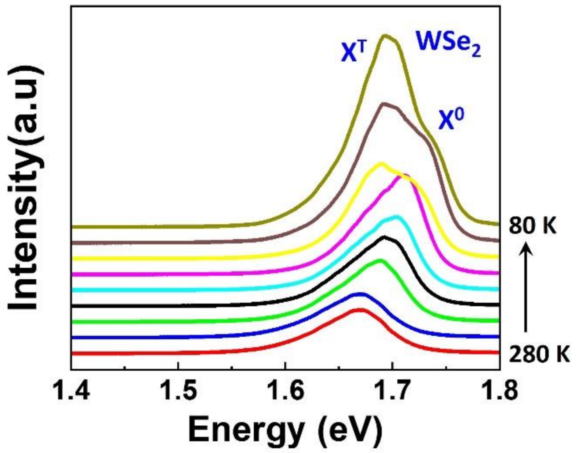

Figure S12. Temperature-dependent PL spectra (280 K to 80 K) of the heterostructures with 1 min-$MoSe_2$ QDs. These spectra were obtained by 633-nm-laser with an excitation power of 20 μW and a 50x lens with 0.35 NA.

Supplementary Figure S13

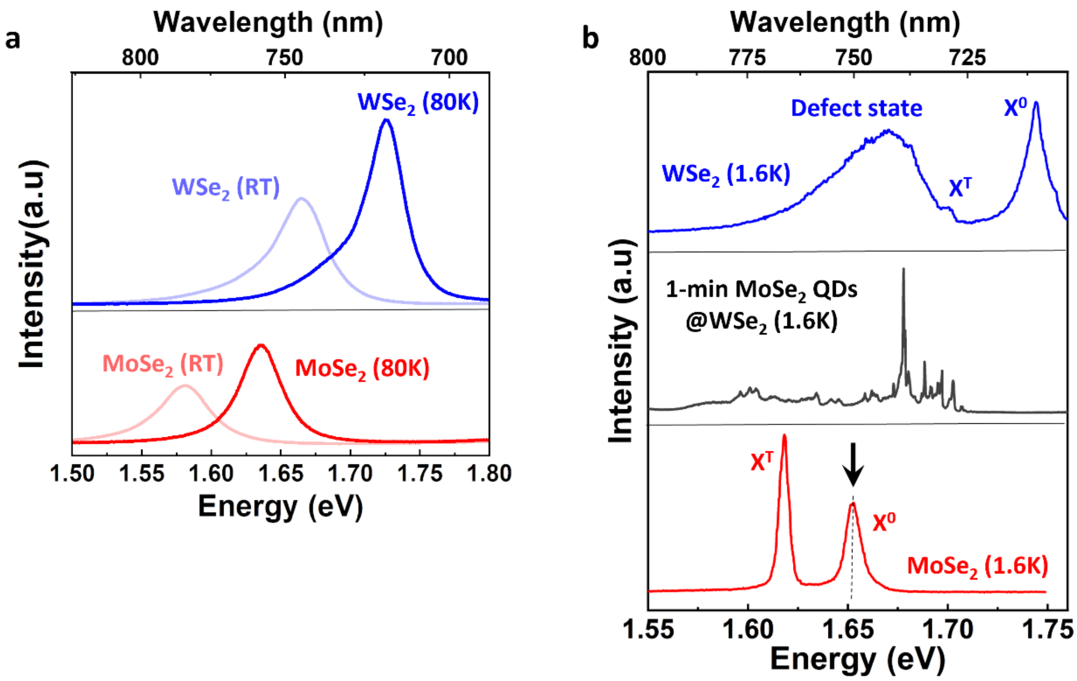

Figure S13. Temperature-dependent PL spectra of reference $MoSe_2$ and WSe_2 monolayers. (**a**) PL spectra of the WSe_2 (top, blue) and $MoSe_2$ monolayers (bottom, red) measured at 80 K (solid) and room temperature (blurry dot) using a 633 nm-laser excitation in a Linkam stage with a liquid nitrogen supply. (**b**) Cryogenic PL spectra of WSe_2 (top, blue) and $MoSe_2$ monolayers (bottom, red) measured at 1.6 K using a 640 nm-laser excitation in a cryostat. For comparison, the cryogenic PL spectrum (middle, black) on the 1-min $MoSe_2$ QDs@WSe_2 heterostructures was added, as shown in **Figure 4b**. The emission peaks in the top and bottom spectra correspond to various excitonic species (neutral excitons, trions, and defect states) in $MoSe_2$ and WSe_2 monolayers, exhibiting broader FWHM values due to homogeneous broadening mechanisms like many-body effects, phonon interactions, and scattering processes within the materials.

Supplementary Figure S14

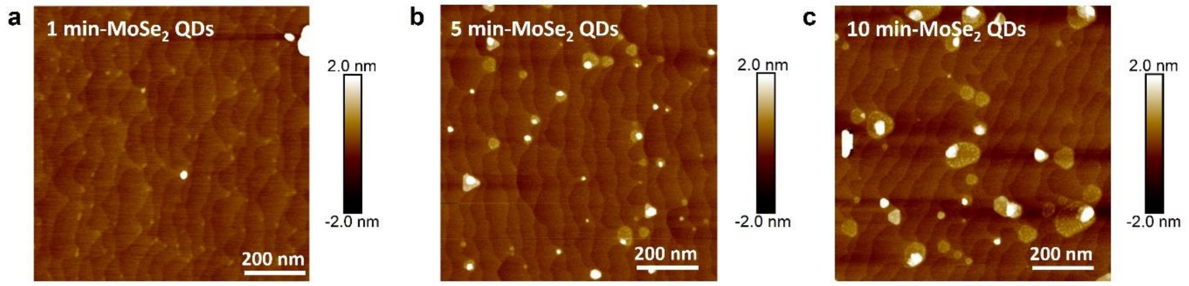

Figure S14. AFM height images of the $MoSe_2$ QDs with different growth times (**a**: 1 min, **b**: 5 min, **c**: 10 min) without the WSe_2 matrix, which were measured on sapphire substrates.

Supplementary Figure S15

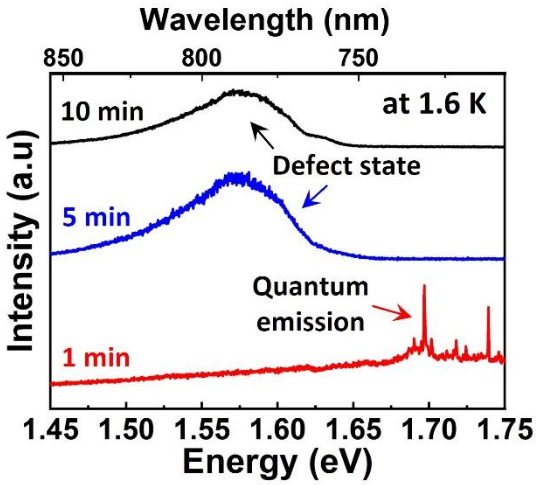

Figure S15. Low-temperature PL spectra of $MoSe_2$ QDs without WSe_2 matrix. Cryogenic PL spectra of the $MoSe_2$ QDs with different growth time of 10 mins (top, black), 5 mins (middle, blue), and 1 min (bottom, red) measured at 1.6 K using a 640 nm-laser excitation in a cryostat.

Supplementary Figure S16

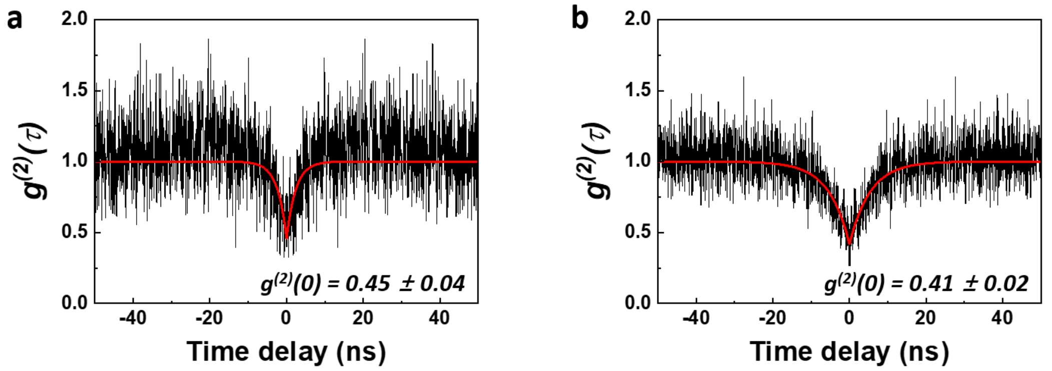

Figure S16. Additional second-order correlation measurement on other points

Supplementary Figure S17

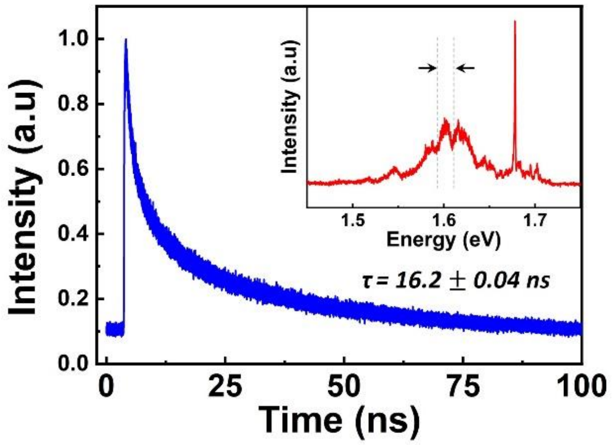

Figure S17. TRPL spectrum for the defect states of the $MoSe_2$ QDs (1.60 eV), shown in Figure 4d, inset.
